# Gambling and financial markets a comparison from a regulatory perspective

**DOI:** 10.3389/fsoc.2022.1023307

**Published:** 2022-11-21

**Authors:** Linus Weidner

**Affiliations:** Department of Sociology, Bergische Universität Wuppertal, Wuppertal, Germany

**Keywords:** gambling, finance, regulation, trading, substitution, stocks

## Abstract

This article discusses similarities between the finance industry and the gambling industry. It considers empirical studies from both fields and compares both industries with regard to possible substitution effects. Afterwards, the current regulatory approach to gambling and financial markets is discussed. Based on this literature review, the author points out that regulators need to acknowledge the fact that both markets possess addictive properties and attract certain risk-seeking individuals. Moreover, the regulators need to find a way to align their fundamentally different objectives to find common solutions to cross-industry problems. Finally, an increased cooperation between (state) authorities is necessary. This cooperation could help to protect traders from developing gambling-related problems, provide significant insights for industry-wide and product-specific regulation and lead to a more informed use of technology for harm prevention purposes. The most important similarities and differences of both markets and the resulting regulatory implications are briefly summarized.

## Introduction

Despite the obvious risk in the financial markets and the speculative nature of trading, the question of whether the financial market possesses any meaningful similarities to the gambling market has largely been neglected from a research perspective [for a perspective on individual behavior, see Fong (2014); for an analysis of the underlying pricing mechanisms, see Levitt (2004); for a cultural studies perspective, see Nicoll (2013)]. On a conceptual and empirical level, Arthur et al. (2016) have shown that differentiation between investment activities, speculation and gambling can provide valuable new insights. While the debate is not entirely new (Borna and Lowry, 1987; Hazen, 1992), the emergence of online discount brokerage services for private clients and the increasing relevance of online gambling markets, make this discussion more essential than ever before.

For the financial markets, the general premise for regulation has been the idea that market agents behave more or less rationally and that “there is no need to protect a fool from his or her investment folly so long as no fraud or manipulation is involved in influencing the investment decision” (Hazen, 1992, p. 987). However, Hazen (1992, p. 1012) already conceded that this assumption might not be correct and that the “laissez-faire approach” to regulation on derivates might lead to increased speculation in the financial market and more gambling (p. 989). Nicoll (2013, p. 390) also makes an argument that “for the past 2 centuries discursive practices of finance sought to foster careful accumulation and prudent decision-making, while those of gambling tended toward undisciplined expenditure and compromised willpower.” She also points out, that nowadays both markets no longer represent opposite moral values and addresses the possible convergence between them. I extend this debate and argue that state authorities could learn from a closer look at the current practices/products in the financial markets and vice versa. This is required to prevent unintended effects of gambling behavior on asset prices (Ji et al., 2021) and to protect the potentially vulnerable group of people who substitute their desire to gamble with financial products that might not be regulated to the same degree (Dorn et al., 2015).

Kumar et al. (2021) argue that in developed countries, 14% of transactions in the stock market are essentially a form of gambling. They also estimate that there is 3.5 times as much gambling in the stock market compared to the regular gambling markets. While both the financial and the gambling market are generally not seen in a favorable light, I argue that certain groups regard the financial market as morally superior compared to the gambling market. This is in line with Skeel (2010) who argues that “evangelicals have been more willing to ‘renegotiate' their stance toward market developments (such as futures contracts) than toward traditional forms of gambling”^1^ The book *Gambling with Other Peoples' Money: How Perverse Incentives Caused the Financial Crisis* even discusses the potential effects that gambling behavior had on the financial system as a whole (Roberts, 2019). Empirical studies that compare the financial markets and gambling are surprisingly scarce, but the evidence shows that both markets possess meaningful similarities (Kumar, 2009; Granero et al., 2012; Dorn et al., 2015). A recent study shows, that modern trading apps such a *Robinhood* use a similar design scheme as digital gambling apps, which arguably leads to an increasing “gamblification” of financial services for private clients (Nicoll and Albarran-Torres, 2022, p. 168). Cox J. et al. (2020) also point out that a sizable group of retail investors meets the criteria for compulsive or problematic gambling. Williams et al. (2022) found in their study with 23,952 Canadian adults that gambling involvement was the strongest predictor for financial speculation. Speculators were also more likely to participate in all forms of gambling with the exception of instant lotteries (Williams et al., 2022). This suggests that both markets attract a similar audience of risk-seeking individuals, which raises the question whether substitution effects between both markets exist.

Before I try to answer this question based on the scientific literature, I begin with a general comparison of the two industries. The first question addresses whether markets are reasonably efficient and can serve as a tool for the accumulation of consistent profits. The general idea behind the efficient market hypothesis (Malkiel and Fama, 1970; Fama, 1998) is that if markets are perfectly efficient, there is no arbitrage between price and value that can be extracted by market participants, because every asset is evaluated correctly at its true intrinsic value^2^. In turn, such a market is only attractive for the (passive) long-term investor^3^. Since we know that humans are not perfect when it comes to financial decision-making (Kahneman and Tversky, 1979; Kahneman et al., 2008) it is unlikely that such a perfectly efficient market exists. A “reasonable speculator” has no incentive to participate in such an extremely efficient market either, since there is no opportunity to beat the general market on a risk-adjusted basis. For the sake of this article, it is most helpful to note that it is fairly likely that markets are only efficient to some degree. Only in this kind of market does speculation become a sensible activity from an economic point of view. Otherwise, all speculative financial activity would essentially equal a pure gamble.

The second important question is whether the individual participating in a form of gambling can use their skills/knowledge to make a profit. Many games that are classified as gambling are fully random by nature. In a lottery, knowledge or skill play absolutely no role. Other games of chance, such as poker or sports-betting are clearly different. They contain an element of luck as well as an element of skill. Which one outweighs the other is finally a regulatory/political issue and defines whether poker is considered gambling or not, for example. This demarcation is also important for taxation purposes. It is worth noting that the best study, at least in the author's opinion, on poker comes to a different conclusion than most regulators (see Fiedler and Rock, 2009). In poker, the players have some influence over how the game is played and over its outcome. There is an element of luck in what cards they are dealt, but players can make the best of those cards by using statistical knowledge, social deduction skills and experience to gain a long-term advantage. What hinders many good players from making a profit are the fees for participating in the game, known as the “rake”. In this regard, the situation is somewhat comparable to the stock market. In both cases, players/traders with higher turnover usually get better conditions (higher rakeback and other benefits or lower transaction fees). Nevertheless, the more professional participants also face disadvantages: in poker, the higher the stakes the tougher the games/opponents, and in the stock market, the bigger market participants face liquidity issues and more regulatory hurdles.

## Substitution effects between the gambling and the financial market

Many studies that compare the gambling and the financial markets do so by looking whether people substitute their desire to gamble with financial products. Dorn et al. (2015) analyzed whether large lotteries and the stock market show any similarities. They found a noticeable and significant negative correlation between the size of the lottery jackpots and the trading volume of private traders who trade in the stock market. Another study uses lottery-related internet search volumes to show that the general sentiment from the gambling market might spill over to the stock market (Chen et al., 2021). Barber et al. (2009) also find a significant decrease in the stock market turnover that corresponded with the legalization of a national lottery in Taiwan. Blau and Whitby (2020, p. 1) also conclude that “countries with more gaming institutions, higher gambling losses per adult, and legalized online gambling have less stable stock prices”. These findings suggest that there might be a substitution effect between both markets. Whether certain individuals are more likely to substitute one activity for the other based on psychological traits or attitudes still needs further research.

I argue that some traders (private and institutional) use the market as a mechanism for gambling. Therefore, it comes as no surprise that some of them “invest” a share of their capital in lotteries if the time is deemed right. Dorn et al. (2015) also show that their findings relate even more to a special portion of the traders. Those who are more likely to play the lotteries generally are also more likely to switch from traditional stock market trading to gambling. For certain other kinds of investment strategies (for example, saving plans), they found no such correlation with lottery participation. Cookson (2018) analyzed the effect of prize-linked savings (PLS) accounts where interest is invested into lotteries. Using a difference-in-difference design, he found that individuals reduced their traditional gambling by at least 3% after PLS accounts were introduced in a country. This indicates that a possible substitution effect between the financial market and the gambling industry works in both directions. This is also shown by a study on numbers gambling in black communities, where locally organized lotteries serve as a financial institution for people whose access to banks and other financial institutions is restricted (Light, 1977). Kumar (2009) shows that lower-income groups tend to prefer “lottery-type” stocks more often, which usually yield lower average returns. Other studies find a “lottery-stock premium” at least for the stock market in Hong Kong (Chan and Chui, 2016). Another study on the Chinese stock market points in a similar direction (Zhu et al., 2021). Nevertheless, this preference seems to be dependent on the general market trend, as. Gong et al. (2021) find a preference for lottery-type stocks in declining markets, while they find no such preference for environments with positive market returns. A disproportional preference for lottery-type assets also seems to exist in the options market (Blau et al., 2016). In summary, these effects, along with a higher propensity to participate in regular lotteries, arguably increases the regressive taxation of lower income groups (Beckert and Lutter, 2008; Kumar, 2009). For regular lotteries, Beckert and Lutter (2008) show that the lower-middle class is effected the most from this.

## Addictive nature of markets

If we extend the argument that financial markets and gambling are similar in some regard, it is worth examining the issue of whether, or to what extent, financial markets have potentially addictive properties, which is shown by many authors (Granero et al., 2012; Fong, 2014; Arthur et al., 2016; Cox J. et al., 2020; Cox R. et al., 2020). In a recent study, Bradley and James (2021) analyzed the content of online forums for people with gambling-related problems, showing that stock market participation is indeed an important subject of discussion. Grall-Bronnec et al. (2017) even conclude from their study with traders who seek help for gambling addiction that “trading and gambling share structural characteristics” and that “excessive trading may be driven by an addictive process”. Shin et al. (2015) compared clinically two groups of people in treatment for pathological gambling issues that originated either from horse-racing or from investing in the financial markets. They found significant differences between both groups with regards to their “clinical and treatment-related features” as well as their sociodemographics. Guglielmo et al. (2016) argue that pathological trading might be a form of addiction. They also created a list of criteria to evaluate whether a person suffers from it (Guglielmo et al., 2016; p. 208).

Based on the author's interviews with people form rehabilitation centers and self-support groups, very few people who lose money in the financial markets join programs designed to cure gambling addictions. Participants in programs such as Gamblers Anonymous (GA) mostly come from a conventional gambling background. This absence of “stock market gamblers” can be explained by two factors: First, people who keep losing money in the market might not realize that they indeed have a gambling problem; second, industry professionals (and private traders to some degree) might fear even more stigmatization and damage to their career prospects if they admit to such issues. This sort of inability to accept a loss (gracefully) and maintain an even head, and instead plunge deeper into the game has been dubbed “being on tilt” by poker players, a saying that has nothing to do with being addicted, *per se*, but primarily refers to an overly emotional response to a series of successive losses or an especially humiliating, and often public, loss. I would argue that traders and fund managers get “tilted” as well. This is quite problematic, as Lo et al. (2005, p. 357) concluded that “one component of successful trading may be a reduced level of emotional reactivity.” Moreover, it is easily imaginable that traders tilt more easily when their own money is on the line compared to trading with third-party funds. Konstantaras and Piperopoulou (2011) also show that retail traders exhibit compulsive behavior when they participate in the market.

Markiewicz and Weber (2013) also find that “investors' gambling risk-taking propensity, measured by the Weber et al. (2002), Domain-Specific-Risk-Taking (DOSPERT) gambling subscale, increases the number of trades made and hence transaction costs, as well as the extent of their day trading”. This suggests that many private investors trade in the financial market with the primary motive of thrill-seeking rather than profit-making (Markiewicz and Weber, 2013, p. 76). A study from the Netherlands estimates that 4.4% of retail investors are compulsive gamblers and another 3.6% show signs of problem gambling (Cox R. et al., 2020). However, Núñez (2017, p. 270) discusses the fact that the medicalization of trading as a gambling disorder usually only relates to “everyday people” rather than people working in banks and other financial institutions. This is an interesting argument and further research on the institutional effects of problematic gambling and trading behavior is certainly needed.

## Examples for the different approaches to gambling and financial regulation

### The industry

If gambling is indeed a part of the stock market, we should also ask what we can learn from gambling regulation. Regulators pursue diverse objectives with their efforts in the gambling market. I will shortly introduce these objectives based on the gambling regulation in Germany. One goal of the regulators is to provide a sufficiently attractive offer so that players do not feel a need to switch to gray/illegal markets to satisfy their desire to gamble (*channeling*). Moreover, regulators attempt to protect young people, provide a fair and reliable market environment, and protect those who are at risk of addiction (see *Glücksspielstaatsvertrag*/*GlüStV*). The billions of tax revenue generated by such gambling markets are, of course, another big motivation for the state. Similarly, the stock market regulators also want a free but fair market environment with profits that make up a large share of the GDP (Mizruchi, 2010; p. 108f).

Sometimes it feels as if regulators are facing an uphill battle. Obviously, this question is not only related to the amount of funding and resources but also to a plethora of specific legal questions and the willingness to prevent industry interests from taking over. In the financial crisis of 2008, industry insiders engaged heavily in creating new regulating authorities. These institutions transferred the logic of the markets to the regulation (Pozner et al., 2010). It was a successful attempt to legitimize trading of complex financial products based on risk valuations that were comprehensible only to former insiders from the investment banking world. This created a system in which former traders and fund managers regulated their own industry (Pozner et al., 2010). The state had no choice but to trust these former industry professionals in their risk evaluations because state authorities lacked the required expertise. Considering the complexity of (electronic) gambling markets, such a state may also arise for the future of gambling markets as well. Therefore, gambling regulators need to find a way to recruit and educate their employees according to their own standards (rather than the industry's) and with enough flexibility to react to changes in the market. In the financial markets, the lines between industry and regulation are blurred further by the central role of rating agencies, which are basically private institutions that rate the credit-worthiness of all publicly traded institutions. Although their role in the financial system has been criticized before (see Poon, 2012), their overall significance has not declined. Such (private) third-party providers of legitimacy/security are a suboptimal solution in any system and should not serve as a role model for the gambling industry. Similarly, Casey (2022) has recently discussed the important role of test houses in the gambling market. These companies assess the conformity of practices in the gambling industry with the regulation and international standards, thereby occupying a similar intermediate position as rating agencies in the financial markets. Since many stakeholders are dependent on these test houses their crucial position in the market is problematic since they “…can affect the extent to which regulation promotes public rather than private interests” (Casey, 2022, p. 166).

### The product

From gambling research, we know that different gambling products have a very different likelihood to cause problematic or even pathological gambling. Hence, gambling products are regulated to a very variable degree. Lotteries, for example, are not as prone to cause tendencies toward problematic play (Binde et al., 2017) and are therefore more easily accessible. Other games, such as slot machines, are regulated more restrictively in terms of availability and turnover in comparison. Especially for slot machines, most of the profits for the casinos/providers are made off a small part of the player base (Fiedler et al., 2019). This is similar in CFD-trading. These contracts for difference are derivates where the buyer bets against the provider of the product that the underlying asset will increase or decrease in price. This means that player protection (in an unrestrictive sense) must be enforced to counteract the inherent conflict of interest. This is why CFDs are heavily regulated. In the US, they are banned entirely, while the German regulator (*Federal Financial Supervisory Authority/BaFin*) decided to reduce potential harm by banning additional payment liabilities for private traders.

This shows that product differentiation also applies to financial products to some degree, but the motivation for regulation is largely different. Investors are generally split into different categories based on their sophistication. This differentiation between private investors, institutional investors and sophisticated investment companies serves a different purpose. It is not the idea of channeling or the prevention of addiction that regulators are concerned with, but the requirement to disclose financial information in a way that is intelligible to each of those groups (Hazen, 1992, p. 1026). The gambling industry, on the other hand, usually allows all customers the same access to all available products. Based on their regulatory goal, the gambling industry found ways to deal with occasions where people start facing serious personal problems as a consequence of their gambling habits. One of the more radical solutions is a (temporary) ban from gambling facilities, which can be initiated by the player himself or the provider. I believe that financial regulators should consider whether the first of those two options might be helpful for certain clients as well.

### The technology

With the increasing legalization of online gambling, there is a discussion about whether electronic/algorithmic surveillance should be introduced to provide automated feedback to players. In the simplest case, this could be a pop-up message that reminds the player to take a break after a certain period of time or tells him how much he lost during the current session. Obviously, regulators need to enforce such measures to ensure that everyone uses them as intended and that all providers are treated equally. While such electronic feedback systems are not necessarily a sufficient solution by themselves (see Bjørseth et al., 2021 for a current meta-analysis), they could also be helpful for (private) traders. In terms of electronic surveillance and the corresponding regulation, the gambling industry might also learn from the financial industry and algorithmic trading (see Eyert et al., 2020). Banks and exchanges already use automatic feedback systems in multiple ways, while trading operations departments monitor all trades and orders from their own personnel and clients. Providers of online gambling services can certainly apply many of these surveillance techniques/systems to monitor (problematic) user behavior. Some concepts from gambling surveillance might also apply the other way around (see Auer and Griffiths, 2015, 2021). Financial institutions that process transactions from gambling are also in a key position to monitor irregular spending that might result from problematic gambling behavior (Swanton et al., 2019). Nevertheless, such practices will only enter the market if some level of regulation is present. In accordance with Abbott (2020, p. 1532), Jonsson et al. (2020) describes the underlying issue as follows:

…*although the technical evolution of gambling increasingly provides the means by which effective duty of care can be provided, this is unlikely to happen unless it is formally mandated and enforced*.

Unfortunately, these measures are unlikely to prevent all kinds of negative outcomes for individuals. Therefore, counseling approaches should also be coordinated across both industries. Pentland and Drosten (1996) worked with two counselors from the West Heidelberg Community Health Center “to identify strategies which might facilitate effective joint casework with gamblers”. While this is by no means sufficient to make general recommendations in this regard, the study might be a starting point for further research on the topic.

The growing cryptocurrencies market brings up another significant regulatory challenge (Brito et al., 2014). Foley et al. (2019), for example, show the significant amount of illegal activities related to the use of cryptocurrencies, a problem that gambling regulators know as well (Potenza et al., 2000; Spapens, 2014; Albanese, 2018). Especially when it comes to money laundering, both markets face significant challenges (Wechsler, 2001; Levi and Reuter, 2006; Buchanan, 2018). Recent research has shown that people are now putting their money into cryptocurrencies, thereby replacing their usual risk-taking preferences from CFD-trading (Pelster et al., 2019), stock market day-trading and sports betting (Delfabbro et al., 2021). This is especially problematic since gambling adverts in sports competitions are increasingly restricted and replaced by adverts for financial and crypto trading-apps (Lopez-Gonzalez and Griffiths, 2018; Newall and Xiao, 2021). Research also shows that higher problem gambling scores are also associated with crypto-trading (Delfabbro et al., 2021). Hence, crypto-trading needs some form of regulation and a clear path toward responsible use regarding gambling. Researchers have also put forth other constructive ideas related to crypto-currencies that integrate well with the general direction of this article, proposing a blockchain-based payment system for the gambling market in Germany (Steinmetz and Fiedler, 2019).

## Conclusions

The aim of this article is to detect similarities in the regulation of financial markets and the gambling industry. Table 1 shows a summary of the most important differences and similarities between both industries and lists the main implications. Research suggests that some parts of the financial markets possess addictive properties (Granero et al., 2012; Fong, 2014; Arthur et al., 2016; Cox J. et al., 2020; Cox R. et al., 2020) and individuals who are active in the financial markets are showing signs of gambling-related problems (Guglielmo et al., 2016; Grall-Bronnec et al., 2017; Cox R. et al., 2020; Mosenhauer et al., 2021). Studies also show that certain gamblers substitute their desire to gamble with a wide range of financial products indicating a direct link between both markets (Dorn et al., 2015; Cookson, 2018).

**Table 1:** Summary of the main findings.

**Important similarities**	**Important dissimilarities**	**Implications**
Both markets can cater to a desire to gamble	While target audiences overlap, they are not equal.	Cooperation and communication between regulators from both industries is required
		
A similar attraction for the player/trader, as highlighted by the diverse substitution effects	Significant differences in the regulatory approach — information provision and fair market access vs. harm reduction and channeling. Differing views on the role of rationality and skill in the decision-making process are closely linked to the different aims of the regulators.	Rethink whether the drastically different approaches to regulation in both markets need to be realigned to some degree.
		
Both markets possess addictive properties	Different ways of dealing with the issue of dependency, both by industry/regulators and affected individuals	A form of (temporary) self-exclusion might be helpful for certain traders. The implementation of cross-industry counseling approaches is necessary (further research required).
		
Product-specific regulations (though with generally different aims). Similarities in the display of certain products	Use of technology and purpose thereof	In certain cases, such as transaction tracking or feedback automation, the use of technology should be discussed between industry stakeholders from both industries. Crypto-trading/-gambling is a new challenge for cross-industry regulation.
		
Problematic role of intermediary organizations		The role of intermediary organizations such as rating agencies and test houses should be reconsidered and other stakeholders' dependency on them must be monitored. Regulators should educate their own personnel, rather than sourcing experts from the industry, to avoid conflicts of interest.

The fundamental reason for regulation in both markets is different, however (see Hazen, 1992). The central task for financial regulators is to make sure that market participants are reasonably informed and sufficient information on companies and products is provided. While access to necessary information should also be guaranteed in the gambling industry (Blaszczynski et al., 2004; p. 311ff), regulators in the gambling industry are primarily concerned with player protection in mind. This evident difference in the approach is a problem because it makes it difficult to find a mutual solution for cross-industry problems such as trading addiction (see Guglielmo et al., 2016). It also prevents the widespread dissemination of counseling services (see Pentland and Drosten, 1996). I conclude from my analysis that market participants who are prone to addiction are currently better protected in a regulated gambling environment as compared to the financial markets where they are still treated as non-existent. While more research in this area is certainly needed, financial regulators should reconsider their approach to regulation in regard to gambling. Nevertheless, I do not argue that regulation in the gambling industry is perfect by any means, which is why I discussed multiple aspects where gambling regulators can learn a lot from regulation in the financial markets as well.

## Implications

Detecting glaring similarities between the financial markets and the gambling industry (see Table 1), one needs to ask whether a non-integrative regulation is sensible. In this article, I argued why this is not the case. While the Reno framework for responsible gambling (Blaszczynski et al., 2004) is discussed critically (Hancock and Smith, 2017), I consent with the framework insofar that cooperation between industry stakeholders is generally beneficial: however, I argue that the cooperation must even extend to regulators in the financial markets in certain cases to better protect vulnerable individuals who substitute activities.

First of all, regulators from both industries need to acknowledge the fact that there are some important similarities between both markets in that both possess addictive properties and attract partially similar audiences of risk-seeking individuals. In a second step, the regulators need to find a way to align their fundamentally different regulatory objectives (see Table 1). Finally, I provided examples where mutual cooperation between regulators from both industries might help to improve regulation at different levels. One aspect is harm prevention and cross-industry counseling in cases where individual gambling problems can't be prevented completely. Another important aspect is the reconsideration and monitoring of the problematic role of intermediary organizations in both markets Especially in regard to the implementation of technology for surveillance, automated feedback, and transaction tracking, the gambling industry and regulation can benefit significantly from an intensified communication with financial institutions. The crypto-currency market is also highly dependent on technological advancement and a new challenge for cross-industry regulation, since crypto-trading platforms are starting to replace sport-betting adverts (Newall and Xiao, 2021).

Any cooperation between the authorities must be mutual, i.e., both parties need to have a legitimate say when they feel that certain standards should be applied to a (new) product or practice in the other market. Whether such cooperation can work effectively on an informal level is difficult to answer at this point. In line with the argument on the implementation of technology in the gambling context (Abbott, 2020, p. 1532; Jonsson et al., 2020), it would certainly be beneficial if this cooperation becomes “formally mandated and enforced.”

## Data availability statement

The original contributions presented in the study are included in the article/supplementary material, further inquiries can be directed to the corresponding author/s.

## Author contributions

The author confirms being the sole contributor of this work and has approved it for publication.

## Conflict of interest

The author declares that the research was conducted in the absence of any commercial or financial relationships that could be construed as a potential conflict of interest.

## Publisher's note

All claims expressed in this article are solely those of the authors and do not necessarily represent those of their affiliated organizations, or those of the publisher, the editors and the reviewers. Any product that may be evaluated in this article, or claim that may be made by its manufacturer, is not guaranteed or endorsed by the publisher.

## Footnotes

^1^Citation taken from the abstract. Available at SSRN: https://ssrn.com/abstract=888184.

^2^Malkiel and Fama (1970) differentiate further between a weak, semi-strong and strong form of the efficient market hypothesis.

^3^Given the assumption that the market offers an adequate risk-reward tradeoff and long-term economic growth is expected.
